# Role of kidney transplantation in long-term cardiac reverse remodeling and interconnecting mechanisms in type 4 cardiorenal syndrome

**DOI:** 10.3389/fneph.2024.1455036

**Published:** 2025-03-06

**Authors:** Jose Luis Salas-Pacheco, Jose Manuel Arreola-Guerra, Ricardo Marquez-Velasco, Israel Perez-Torres, Sergio Casarez-Alvarado, Giovanny Fuentevilla-Alvarez, Verónica Guarner-Lans, Randall Cruz-Soto, María Elena Soto

**Affiliations:** ^1^ Cardiology Department, Centenario Hospital Miguel Hidalgo, Aguascalientes, Mexico; ^2^ UNAM Master’s and Doctoral Program in Medical, Dental and Health Sciences UNAM, Mexico City, Mexico; ^3^ Nephrology Department, Centenario Hospital Miguel Hidalgo, Aguascalientes, Mexico; ^4^ Immunology Department, Instituto Nacional de Cardiología Ignacio Chavez, Mexico City, Mexico; ^5^ Cardiovascular Biomedicine Department, Instituto Nacional de Cardiología Ignacio Chavez, Mexico City, Mexico; ^6^ Physiology Department, Instituto Nacional de Cardiología Ignacio Chavez, Mexico City, Mexico; ^7^ Research Direction, Instituto Nacional de Cardiología Ignacio Chavez, Mexico City, Mexico; ^8^ Cardiovascular Line Department, Centro Médico ABC Sur, Mexico City, Mexico

**Keywords:** cardiorenal syndrome, left ventricle hypertrophy, reverse cardiac remodeling, kidney transplantation, echocardiography, fibroblast growth facctor-23, microRNA

## Abstract

**Background:**

Type 4 cardiorenal syndrome (CRS) involves cardiovascular alterations caused by chronic kidney disease (CKD). Fibroblast growth factor-23 (FGF23), carboxy-terminal propeptide of procollagen type I (PIP), and parathyroid hormone (PTH) have been proposed as biomarkers of pathological cardiac remodeling in CKD. In contrast, it has been suggested that MicroRNA 221 has a cardioprotective role. Available evidence shows that, 12 months after kidney transplantation (KT), type 4 CRS reverts in only half of the patients.

**Objective:**

To assess long-term cardiac reverse remodeling after KT and its association with FGF23, PIP, and PTH levels.

**Methods:**

Patients with end-stage renal disease were assessed before and 28 months after KT using FGF23, PIP, and PTH serum concentrations and transthoracic echocardiography.

**Results:**

Fifty-three patients were followed for 28 months after KT. All the patients showed cardiac abnormalities upon inclusion. A follow-up assessment showed a reduction in left ventricle (LV) mass (121 ± 48 vs. 65 ± 14 gr/m^2^) and left atrial volume (46 vs. 30 ml/m^2^). The LV ejection fraction (53 vs. 63%), LV global longitudinal strain (-15.9 vs.-19.4%), and LV diastolic function improved. miR-221 expression increased after KT (8.73 RIQ= 3.7-25 vs. 40.16 RIQ= 24-223, p=0.001) and was correlated with the Ee´ratio (r= -0.32, p= 0.02). Multivariate analysis showed that post-KT LV mass was determined by pre-KT LV mass, serum Cr level, post-KT PIP, and hypertension (R^2^ = 0.65, F=12.1, p=0.001).

**Conclusions:**

Contrary to other evidence, this study demonstrated that type 4 CRS is reversible over the long term. This is a paramount finding because KT normalizes cardiac structure and function independently of the severity of basal cardiac abnormalities.

## 1 Introduction

Cardiorenal syndrome (CRS) encompasses the interactions between the heart and kidneys, where acute or chronic dysfunction in one organ can induce dysfunction in the other (1, 2).

Type 4 CRS involves cardiovascular alterations caused by chronic kidney disease (CKD). The cardiac phenotype shows alterations induced by pressure and volume overload (3). The main findings are cardiac chamber dilatation, LV hypertrophy, LV diastolic dysfunction that is almost universal, and LV systolic dysfunction, with a prevalence ranging from 16 to 28% (4, 5). Hystologically, Type 4 CRS is characterized by a structural disarray of cardiomyocytes, severe myocyte hypertrophy, and increased cardiac interstitial collagen deposition (6).

The decrease in glomerular filtration rate triggers a surge in toxic substances known as uremic toxins. Fibroblast growth factor 23 (FGF23) is associated with pathological cardiac remodeling and an increased risk of adverse cardiovascular events (7–9). *In vitro* studies have shown that high levels of FGF23 modify the phenotype of smooth vascular muscle cells (VSMCs) from contractile to synthetic by acting on its specific receptors FGFR1 and Erk1/2. This change was mediated by the downregulation of miR-221/222, which increased the expression of MAP3K2 and PAK1. Moreover, miR-221/222 transfection recovered the contractile phenotype of VSMCs. Additionally, infusion of recombinant FGF23 in rats increased vascular wall thickness and VSMCs showed a synthetic phenotype with reduced miR-221 expression. Ex vivo studies on aortic rings from patients with CKD demonstrated that elevated FGF23 levels increased arterial wall stiffness and were associated with increased pulse wave velocity and reduced plasma levels of miR-221/222 (10).

Some studies have suggested that parathyroid hormone (PTH) can induce LV hypertrophy (11, 12) in a similar manner as FGF23; however, the evidence is contradictory.

MicroRNAs (miRs) are small non-coding RNAs measuring between 20 and 25 nucleotides in length that are involved in the regulation of gene expression. Recently, miR-221 was reported to exhibit cardioprotective and antifibrotic properties. Specifically, it promotes the survival of cardiomyocytes during stress conditions and suppresses excessive collagen production (13–16).

Histological analysis of the heart in patients with CKD shows a high amount of interstitial collagen, which deteriorates the mechanical properties of the heart and promotes the development of heart failure (17). Endomyocardial biopsy is the standard method for measuring interstitial collagen; nevertheless, its implementation in routine clinical practice is not feasible (18). However, the serum concentration of the carboxy-terminal propeptide of procollagen type I (PIP) serves as a reliable and non-invasive proxy for assessing the quantity of interstitial collagen (19–21).

**Figure 1:**
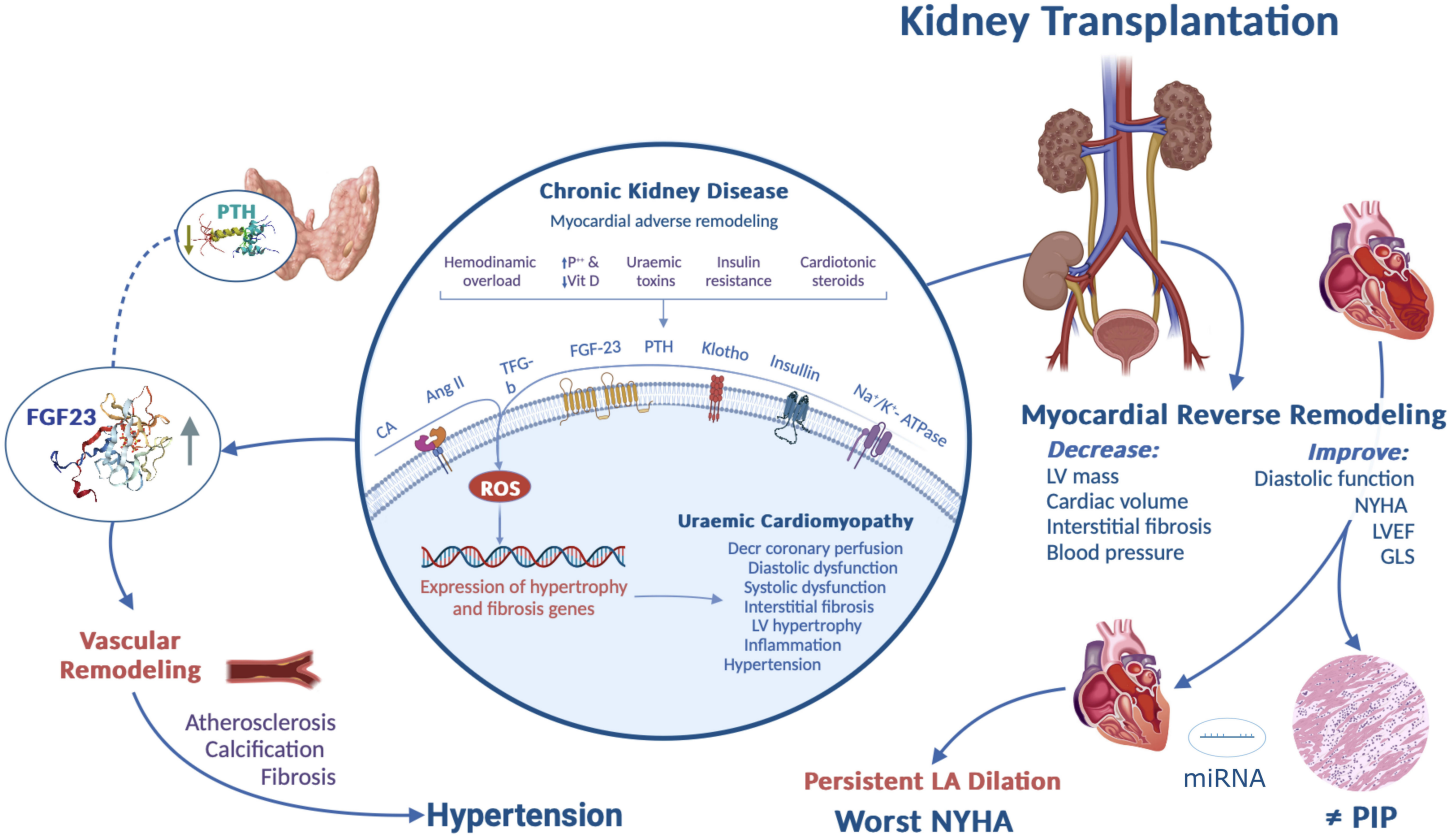
Graphical abstract. Cardiorenal syndrome, which encompasses the complex interplay between cardiac and renal dysfunction, has significant physiological implications in various organs and systems.

Kidney transplantation (KT) has proven to be highly effective in restoring the uremic environment and relieving hemodynamic overload caused by CKD (**Figure 1**). However, recent studies have shown that substantial cardiac abnormalities prevail in approximately half of the patients in the short term (22–24). Therefore, this study aimed to evaluate the extent of long-term reverse cardiac remodeling following KT using echocardiography and to explore its relationship with serum levels of FGF23, PIP, PTH, and miR-221.

## 2 Materials and methods

### 2.1 Study population

This dynamic prospective cohort study was conducted between March 2019 and November of 2022, and included both male and female patients aged > 18 years who were diagnosed with CKD at K-DIGO stage V, irrespective of whether they were receiving renal replacement therapy, and who were approved by the institutional transplant committee to receive KT. Patients with diabetes mellitus were not eligible for inclusion. Patients who did not undergo KT after the initial evaluation, those who experienced graft loss, and those lost to follow-up were excluded. On the day preceding the surgical intervention, transthoracic echocardiography, and blood samples were collected. Subsequent to the procedure, telephone follow-up was conducted every six months. At 28 months follow-up, additional transthoracic echocardiography and blood samples were obtained. Blood pressure was measured noninvasively using an aneroid sphygmomanometer (WA7670-10, WA, Inc.), following the World Health Organization technical specifications for non-invasive blood pressure measuring devices with a cuff. Graft function was assessed using serum creatinine (Cr) levels, and creatinine clearance (CrCl) was estimated using the CKDEPI equation.

### 2.2 Cardiorenal syndrome operational definition

Type 4 CRS was considered present when patients had alterations in LV geometry, LV dilatation, LV hypertrophy, or abnormalities in systolic or diastolic LV function (25).

### 2.3 Transthoracic echocardiography

Transthoracic echocardiography was performed using a Philips Epiq 7 ultrasound and a 2–5 mHz sectorial transducer following the recommendations of the American Society of Echocardiography (ASE) (26). Left ventricle (LV) mass was calculated using the modified Devereux equation, and chamber volumes were measured using Simpson´s method. The left ventricular ejection fraction (LVEF) was determined using the biplane disc method. Diastolic function was evaluated using pulsed-wave Doppler of transmitral flow, tissue Doppler mitral annulus velocities, left atrial (LA) volume, and tricuspid regurgitation maximum gradient (27). Pulmonary artery systolic pressure (PASP) was determined by adding the right atrial pressure (estimated by the diameter and collapsibility of the inferior vena cava) to the maximum gradient of tricuspid regurgitation. The left ventricular global longitudinal strain (GLS) was obtained from an average of 16 segments using the QLab software (version 13.0) and the LV auto-strain tool (Philips, Andover MA).

### 2.4 Blood sample collection

Blood samples (12 ml) were collected from each patient by venipuncture. The samples were then centrifuged at 2500rpm for 15 min at 4°C without any additives. The resulting components were then placed in 1.5 ml Eppendorf tubes and stored at -80°C until biomarker quantification and analysis could be performed.

### 2.5 Blood sample analysis

For the FGF23, PIP, and PTH ELISA tests, the manufacturer’s recommendations were followed. Biological samples of 500 µL were used for each assay. FGF23 levels were quantified using the Human FGF23 monoclonal antibody and R&D Systems reagent (Minneapolis, MN, USA). The kit featured a lower detection limit of 3 pg/ml and detection range of 3-8000 pg/ml. For PIP assessment, a reagent sourced from MyBioSource (San Diego, CA, USA) was used, with a detection range of 25-2000 ng/ml and sensitivity of 5 ng/ml. Serum PTH levels were determined using a reagent from Abbexa (Texas, USA), with a detection range of 3.13-800 pg/ml and a sensitivity of 1.88 pg/ml.

Serum Cr concentration was quantified using standardized dry chemistry (Vitros 4600; Ortho Clinical Diagnostics). Hemoglobin levels were assessed using a hematology analyzer (DxH900 Workcell Solution; Beckman Coulter, Inc.).

### 2.6 Quantification of miRNA-221-5p

Total RNA was purified from plasma samples using the miRN easy Serum/Plasma Kit (Qiagen) following the manufacturer’s instructions. The pulsed reverse transcription reaction was performed in serum to obtain cDNA of miR-221-5p, using the specific primers for the mature forms, through the TaqMan miRNA RT Kit (TaqMan^®^ Advanced miRNA cDNA, Synthesis Kit, Applied Biosystem, Foster City, CA, USA, Catalog Number A28007). miR-221-5p (hsa-mir-221-5p) was quantified using a commercial system kit (TaqMan gene expression assay) for miRNA using the CFX96 real-time PCR system (Bio-Rad). The expression levels were measured in duplicate and normalized to the endogenous gene miR-16 (hsa-mir-16). Relative quantification was performed using the following formula: 2−ΔΔCt (28).

### 2.7 Ethical statement

This study was approved by the Institutional Ethics Committee (protocol number CEI/52/CI/32/18) and conducted in accordance with the Declaration of Helsinki. The study protocol followed the Strengthening the Reporting of Observational Studies in Epidemiology (STROBE) guidelines. Written informed consent was obtained from all patients.

### 2.8 Statistical analysis

We estimated the sample size with 95% confidence and 80% power, which gave us a sample size of 55 patients, and used the formula for the difference in means. The variable distributions were evaluated using the Kolmogorov-Smirnov test. The association between qualitative variables was assessed using the chi-square test. Differences between groups were identified using Student’s t-test or the Mann-Whitney U test. Correlations were evaluated using Pearson’s or Spearman’s correlation coefficient. Differences between the paired data before and after KT were assessed using the paired t-test or Wilcoxon signed-rank test. Differences between the LA volume categories (normal volume, mild dilation, and moderate dilation) were evaluated using the Kruskal–Wallis test. Predictors of persistent arterial hypertension after KT were assessed using a logistic regression analysis. Multiple regression analysis was used to identify the variables associated with post-KT LV mass. Both multivariable models included predictor variables with a statistical significance of < 0.1 in the bivariate analysis. Differences were considered significant when the p-value was less than 0.05 bilateral. The statistical analysis was conducted using the R software (version 4.2.3) with R Studio interface (version 2023.03.0 + 386).

## 3 Results

### 3.1 Pre-transplant

**Table 1** presents the demographic characteristics. We included 53 patients, with a mean age of 34 ± 11 years. Sixty-three percent of the patients were male, and CKD etiology was idiopathic in most cases. Six patients did not receive renal replacement therapy (RRT) at admission, and the remaining patients were on hemodialysis three times per week. The median RRT lapse duration was 10 months. Hypertension was present in 75% of the patients, and all were poorly controlled despite a minimum of three antihypertensive drugs. Twenty-three patients were excluded, primarily because they were lost to follow-up.

**Table 1:** General demograhic characteristics.

Variable	n= 53
Pre-Transplant	
Female	20 (37)
Male	33 (63)
Age (years)	34 (12)
NYHA FC	
I	7 (14)
II	37 (70)
III	8 (16)
Hypertension	40 (76)
Weight (Kg)	69 (13)
Unknown Etiology	42 (78)
Months on HD	10 (6-18)
CKD etiology	
Idiopatic	38 (72)
Renal hypoplasia	7 (14)
Glomerulonephritis	5 (9)
APCKD	3 (5)
Post-Transplant	
Re-Hospitalization	25 (28)
Hypertension	11 (21)
Tacrolimus (mg)	5 (2)
MMF (gr)	2 (1-2)
Prednisone (mg)	5 (2)
Donnors	
Age (years)	40 (31-50)
Female	31 (51)
Male	22 (43)
Live related	38 (71)
Live non related	11 (21)
Cadaveric	4 (8)

APCKD, Adult polycystic kidney disease; FC, functional class; HD, hemodialysis. The values are expressed in mean (±SD), median (RIQ 25-75), and absolute numbers (%).

Initial echocardiographic investigation showed that concentric hypertrophy was the most common pattern of left ventricular (LV) geometry, accounting for 48% of cases. The mean LVEF was 53% ± 11; however, 36% of the patients had a value of < 50%. The majority of patients (62%) had reduced LV GLS, with a mean value of -13.5%. Abnormal LV diastolic function was observed in 81% of the patients, and 34% had evidence of increased LV filling pressure. Left atrial dilatation was present in 80% of patients, with a mean volume of 46 ± 16 mL/m^2^.

The RRT duration before KT affected cardiac structure and function. The findings revealed that LV mass (r^2^ = 0.40, p = 0.03), LV volume (r^2^ = 0.30, p = 0.02), LVEF (r^2^ = 0.27, p = 0.04), and GLS (r^2^ = 0.32, p = 0.01) were worse in patients undergoing hemodialysis for longer periods. This was similar to diastolic function, as indicated by the E/e’ ratio (r2 = 0.37, p = 0.006).

**Figure 2:**
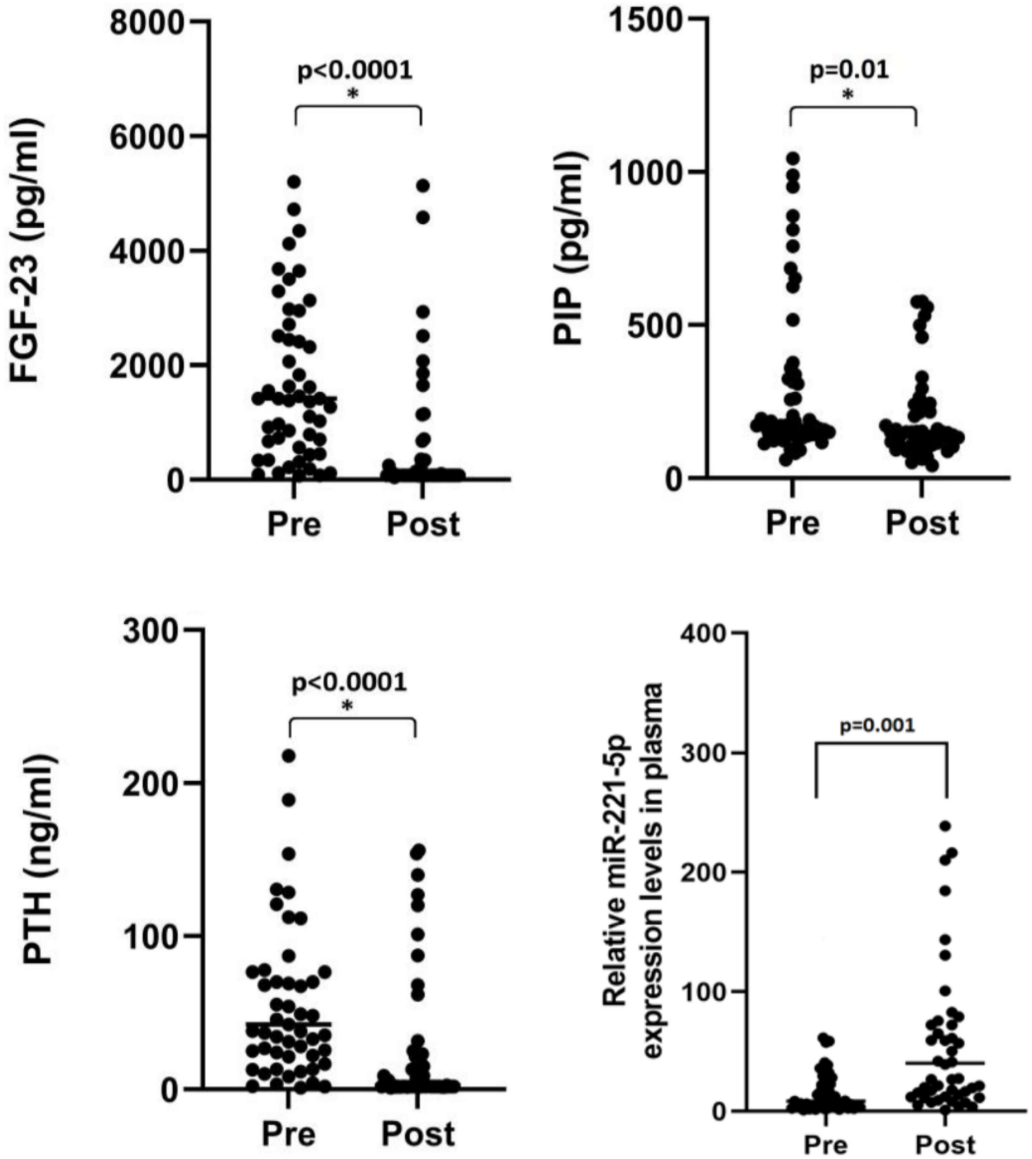
Changes in serum biomarker levels after kidney transplantation.

All patients had elevated FGF23 and PIP levels (**Figure 2**). Surprisingly, the PTH levels were lower than expected (42 pg/ml; RIQ: 22-78). Patients with higher FGF23 levels exhibited a higher LV mass (r= 0.4, p= 0.001) and had been receiving RRT for an extended period (r= 0.3, p= 0.04). PTH level was not associated with structural or functional cardiac abnormalities. Examination of the interactions between biomarkers revealed that individuals with normal or inhibited PTH levels had increased FGF23 concentrations (H= 5.9, p= 0.049).

### 3.2 Post-transplant

After KT, patients were followed up for an average of 28 ± 6 months. The New York Heart Association (NYHA) functional class scores improved considerably (**Table 2**). Additionally, at six months, all patients experienced resolution of anemia. One-third (28%) of the patients were hospitalized within the first three months following KT because of urinary tract infection; however, this did not correlate with adverse long-term outcomes.

**Table 2:** Change in clinical and biochemical variables, and biomarkers after kidney transplantation.

Variable	Pre-KT n= 53	Post- KT n= 53	p
NYHA I	7 (14)	31 (58)	0.001
NYHA II	37 (70)	22 (42)	0.001
NYHA III	8 (16)		
Hypertension	40 (75.5)	11 (20)	0.001
Systolic BP	142 (16)	114 (8)	0.001
Diastolic BP	88 (10)	72 (6)	0.001
Creatinine	7.9 (1.3)	1.2 (0.3)	0.001
Hemoglobin (gr/dL)	9.2 (2.3)	13.6 (1.8)	0.001
FGF-23 (pg/ml)	1418 (2953-6706)	78 (78-345)	0.001
PIP (pg/ml)	171 (143-338)	151 (108-243)	0.003
PTH (ng/ml)	42 (22-78)	2.5 (1.8-21)	0.001

New York Heart Association= NYHA, KT= Kidney Transplantation.

The prevalence of hypertension decreased significantly, and only 20% of patients had hypertension. However, all patients achieved their blood pressure goals using a single drug (46 patients received an angiotensin receptor antagonist, six received a calcium channel blocker, and one received a beta-blocker). The only variable associated with persistent hypertension was the pre-KT FGF23 level (X^2^ = 12.1, R^2^ = 0.3, p=0.032). Surprisingly, the duration of RRT did not correlate with post-KT cardiac structure and function or with persistent hypertension.

The long-term graft function demonstrated a mean serum Cr level of 1.22 mg/dL and CrCl of 65 mL/min/1.73 m^2^. Notably, CrCl was below 60 mL/min/1.73 m^2^ in 43% of the patients, but it was not less than 30 mL/min/1.73 m^2^. Among the variables analyzed, only donor age was associated with lower CrCl (r= -0.29, p= 0.03).

**Figure 3:**
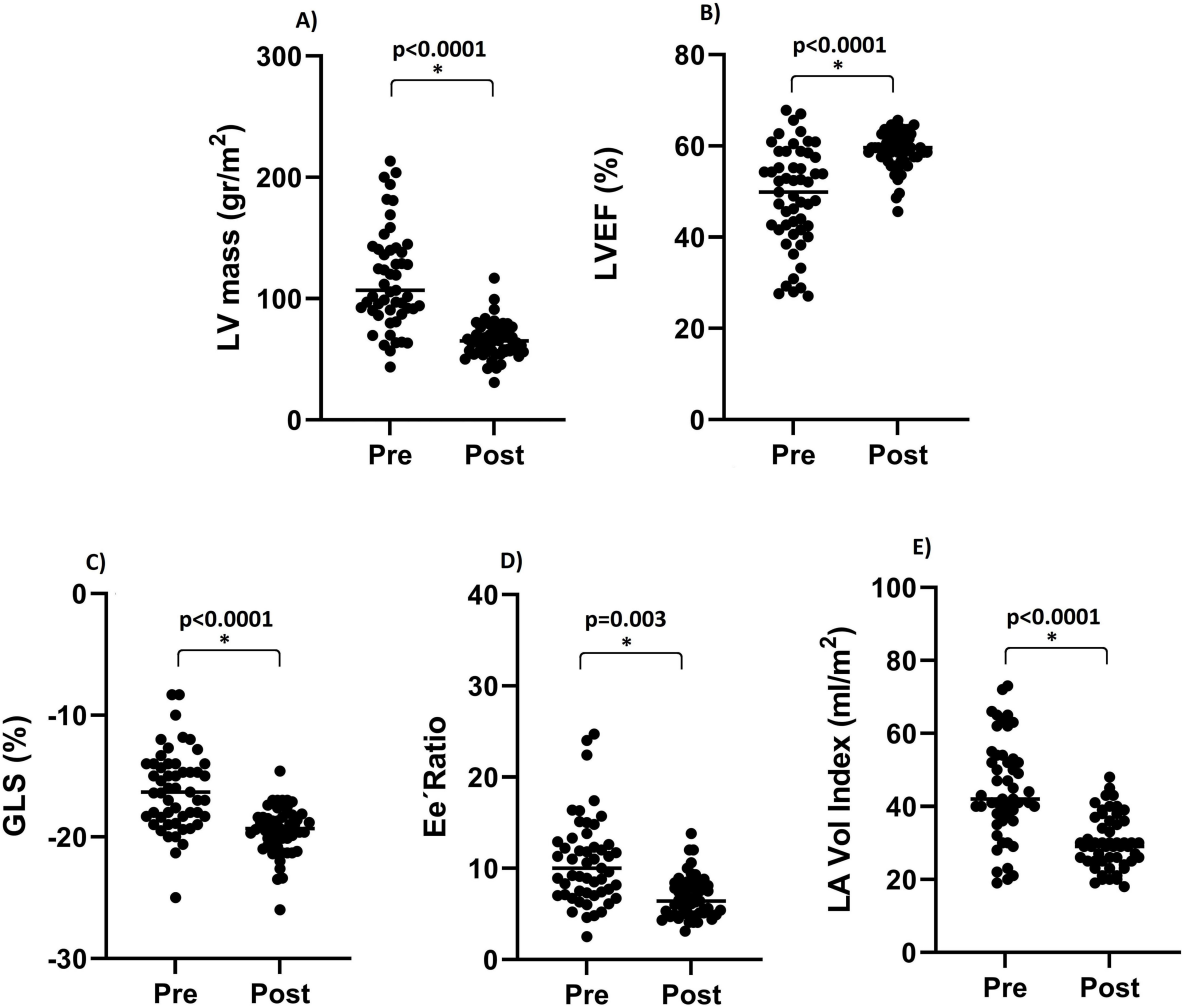
Changes in echocardiographic parameters after kidney transplantation. The figure shows the change in left ventricle mass **(A)**, left ventricle ejection fraction **(B)**, left ventricle global longitudinal strain **(C)**, Ee´ ratio **(D)**, and left atrial volume **(E)** after kidney trcasplantarion. GLS, global longitudinal strain; LA, left atrial; LV, left ventricle.

**Figure 4:**
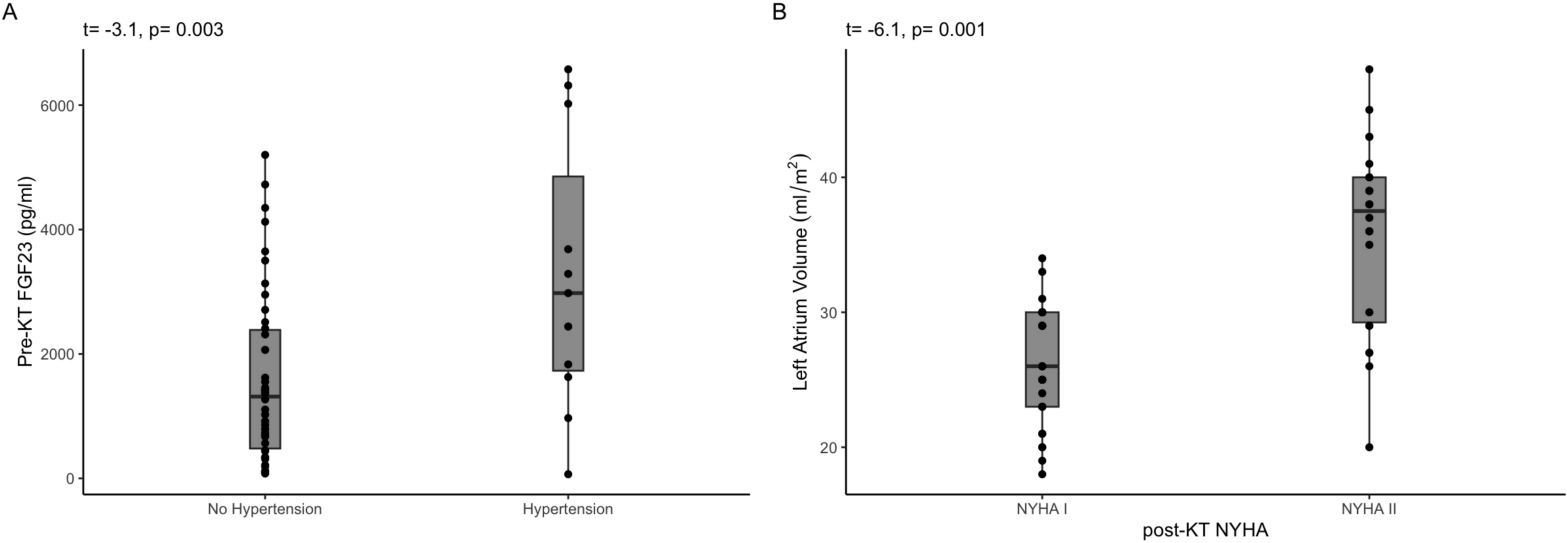
**(A)** displays the distribution of post-KT FGF23 levels based on the hypertension status. **(B)** shows that the patients with larger LA volumes had the worst NHYA class.

According to echocardiographic data, all patients demonstrated a decrease in both the volume of the cardiac chambers and LV mass (**Table 3**). In terms of LV systolic function, LVEF and GLS increased by 10 and 3 percentage points, respectively. Notably, the LVEF was >50% in all patients. The pre- and post-transplant echocardiographic findings are presented in **Table 3** and **Figure 3**, respectively. Despite a significant decrease in LA volume, six patients showed mild LA dilatation (LA volume index 34-38 ml/m^2^), and nine patients exhibited moderate LA dilatation (LA volume index 39-48 ml/m^2^). Individuals with a dilated LA had greater LV mass (r= 0.43, p= 0.001) and worse diastolic function (r= 0.31, p= 0.023). Similarly, **Figure 4** shows that patients with NYHA class II had a significantly higher LA volume than those with NYHA class I (35 ± 7 vs. 25 ± 5 ml/m2, t= 6.1, p= 0.001). Despite multiple baseline cardiac alterations, reverse remodeling after KT significantly improved cardiac size and function. **Table 4** shows the population according to basal LVEF; it is evident that even in the sickest patients, LV systolic function is normalized after KT.

**Table 3:** Change in echocardiographic parameters after kidney transplantation.

Variable	Pre-KT n= 53	Post- KT n= 53	p
LV septum EDD (mm)	11.9 (2)	9 (1.4)	0.001
Relative wall thickness	0.51 (0.1)	0.43 (0.08)	0.001
LV mass (gr/m^2^)	121 (48)	65 (14)	0.001
LV normal geometry	2 (3)	26 (49)	0.001
Concentric remodeling	21 (39)	27 (51)	0.038
concentric hypertrophy	25 (47)		
Eccentric hypertrophy	5 (9)		
LV ED Vol (mL)	123 (39)	95 (20)	0.001
LV ES Vol (mL)	60 (28)	35 (8.4)	0.001
LVEF (%)	53 (11)	63 (4.4)	0.001
LV GLS (%)	-15.9 (3.5)	-19.4 (1.98)	0.001
Ee´ ratio	10 (7-14)	6.8 (2.2)	0.001
LA Vol (ml/m^2^)	46 (16)	30 (7.8)	0.001
PASP (mmHg)	37 (13)	23 (4)	0.001

BP, blood pressure; DD, diastolic dysfunction; DF, diastolic function; EDD, end-diastolic diameter; ED, end-diastolic; ES, end-systolic; GLS, global longitudinal strain; Ind, index; LA, left atrial; LV, left ventricle; PASP, pulmonary artery systolic pressure; Vol, volume.

**Table 4:** Biochemical and echocardiographic characteristics grouped according the basal LVEF and after KT.

Variable	Pre-KT (n=53) Median (min-Max)	Post-KT (n=53) Median (Min-Max)	p
<40% LVEF (n= 7)			
LV septum (mm)	13 (10-16)	9 (7-11)	0.01
LV mass (gr/m^2^)	145 (90-236)	65 (54-117)	0.01
LV ED Vol (mL)	168 (68-187)	100 (60-146)	0.01
Ee´ ratio	16.2 (7-37)	8 (4.4-12)	0.01
LA Vol (ml/m^2^)	54 (23-72)	30 (19-57)	0.01
PASP (mmHg)	44 (38-46)	22 (20-34)	0.01
FGF23 (pg/mL)	1619 (452-4723)	78 (78-2068)	0.06
PIP (pg/mL)	325 (124-990)	133 (93-919)	0.01
PTH (ng/mL)	54 (1-87)	9 (2-22)	0.04
LVEF (%)	33.7 ± 2.1	62.04 ± 1.7	0.0001
GLS (%)	-10.91 ± 3.6	-20.14 ± 2.7	0.0001
40-50% LVEF (n=12)			
LV septum (mm)	13 (10-16)	10 (7-12)	0.003
LV mass (gr/m^2^)	143 (97-213)	66 (57-91)	0.002
LV ED Vol (ml)	146 (93-178)	105 (86-137)	0.003
Ee´ ratio	12 (6.7-24)	6 (4.1-8.9)	0.004
LA Vol (ml/m^2^)	50 (28-92)	29 (18-43)	0.002
PASP (mmHg)	42 (25-75)	24 (20-30)	0.003
FGF23 (pg/mL)	825 (67-6022)	78 (78-675)	0.008
PIP (pg/mL)	171 (92-1043)	156 (63-836)	0.8
PTH (ng/mL)	32 (8.3-76.5)	47 (1.9-275)	0.7
LVEF (%)	44 ± 2.3	63 ± 4.5	0.0001
GLS (%)	-14.01 ± 2.1	-19.12 (2.4)	0.0001
>50% LVEF (n=34)			
LV septum (mm)	11 (8-21)	9 (7-12)	0.0001
LV mass (gr/m^2^)	95 (44-275)	63 (31-99)	0.0001
LV ED Vol (ml)	106 (63-246)	94 (50-142)	0.001
Ee´ ratio	8.8 (2.5-22.4)	6.7 (3-14)	0.003
LA Vol (ml/m^2^)	41 (19-89)	30 (20-45)	0.0001
PASP (mmHg)	30 (20-97)	22 (18-40)	0.0001
FGF23 (pg/ml)	1418.6 (7-7133)	78 (46-6027)	0.0001
PIP (pg/ml)	164 (60-1241)	151 (42-1071)	0.01
PTH (ng/ml)	47 (1.9-275)	2.1 (1.2-156)	0.0001
LVEF (%)	60 ± 6.08	64 ± 4.8	0.0001
GLS (%)	-17.6 ± 2.4	-19.3 ± 1.8	0.0001

LVEF, Left ventricle ejection fraction; EDD, end-diastolic diameter; ED, end-diastolic; ES, end-systolic; GLS, global longitudinal strain; LA, left atrial; LV, left ventricle; PASP, pulmonary artery systolic pressure; Vol, volume; PIP, Procollagen I propeptide; PTH, Parathyroid hormone.

Regarding LV mass after KT, multivariable analysis identified pre-KT LV mass (r^2^ = 0.15, p=0.001), serum Cr (r^2^ = 16.6, p=0.001), post-KT PIP (r^2^ =-0.015, p=0.01), and hypertension (r^2^ = 8.5, p=0.01) as predictor variables. The overall regression was significant (r^2^ = 0.65, p=0.001).

A significant decrease in the FGF23, PIP, and PTH levels was observed at 28 months (**Figure 2**). Pre-KT FGF23 levels were associated with persistent hypertension (X^2^ = 12.1, R^2^ = 0.3, p=0.032). Post-KT PIP levels were weakly associated with LV mass. The expression of miR-221 was significantly increased after KT (8.73 RIQ= 3.7-25 vs. 40.16 RIQ= 24-223, p=0.001) and was negatively correlated with the Ee´ratio (r= -0.32, p= 0.02), a parameter used for diastolic function assessment.

## 4 Discussion

The specifics of the pathophysiology associated with type 4 CRS are not yet fully understood, although they are believed to be the result of several interrelated factors such as neurohormonal activation, inflammation, oxidative stress, and impaired endothelial function (29). Individuals with CKD exhibit a heightened risk of cardiac-related death, which is 10–20 times greater than that in matched controls. Thus, CKD patients die prematurely from cardiovascular diseases rather than from renal dysfunction (30). After renal transplantation, mortality of cardiovascular origin remains high; up to 30% of patients die of cardiovascular disease (31).

The patients included in this study were mostly young and without other chronic diseases such as diabetes mellitus. Thus, this population may reflect the behavior of cardiovascular disease after kidney transplantation, isolated from its interaction with other chronic pathologies. That is, it is a model of cardiorenal syndrome, without the post-transplant influence of the main risk factors for major cardiovascular events. According to the results of this study, renal transplantation leads to significant improvements in both structural and functional cardiac anomalies regardless of pre-existing abnormalities. Discrepancies with other studies may reflect the sustained deleterious effects of other comorbidities on the cardiovascular system.

In systemic diseases involving the heart or in primary cardiac diseases characterized by pressure overload, the myocardial interstitium experiences a specific type of cardiac fibrosis called reactive or diffuse fibrosis, which is characterized by the diffuse deposition of cross-linked collagen in interstitial and perivascular areas. In advanced stages, myocytes are surrounded by widespread fibrous tissue, resulting in elevated LV filling pressure, symptoms of heart failure, and poor response to standard treatment (32, 33). Histologically, in CKD, myocytes display structural disarray and severe hypertrophy with a lower proportion of interstitial fibrous tissue than in other cardiac diseases (34). This minor relative amount of fibrous tissue may explain the weak correlation between the PIP levels and LV mass. This observation suggests that the reduction in myocyte size, rather than a decrease in interstitial volume, primarily accounts for the diminution in LV mass. This phenomenon may be the basis for the impressive improvement in LV systolic and diastolic functions after KT.

Previous studies using echocardiography that assessed cardiac reverse remodeling 12 months after KT found that LV hypertrophy and systolic dysfunction persisted in 60% of patients (22–24). Similar results were observed in patients with hypertension and aortic stenosis despite receiving treatment (35, 36). In contrast, Franz et al. observed that in hypertensive patients, after 32 months of optimal blood pressure control, LV mass normalized in >90% of the patients (37). Similar to Franz et al., we observed a significant reduction in LV mass 28 months after KT. This demonstrates that resolution of LV hypertrophy is a long-term phenomenon. Pressure overload is not the only mechanism that triggers LV hypertrophy. The presence of multiple comorbidities, including diabetes, obesity, and obstructive sleep apnea, has been documented to play independent or synergistic roles in the development of LV hypertrophy (38). Long-term follow-up and absence of other comorbidities are critical factors that could contribute to our findings.

Studies have suggested that miR-221 plays an important role in the regulation of cell survival in cardiovascular diseases. Furthermore, decreased miR-221 expression in the myocardium is associated with severe cardiac fibrosis in HF patients. It has been proposed that miR-221 enhances cardiomyocyte survival by inhibiting apoptosis and autophagy through downregulation of total P53 protein expression (39). Preclinical studies have indicated that miR-221 reduces cardiac fibrosis in chronic kidney disease (CKD) by suppressing the expression of thrombospondin 1 and transforming growth factor B1 (40). Myocardial fibrosis is an important pathophysiological process that contributes to diastolic and eventually systolic dysfunction by increasing myocardial stiffness and reducing LV pumping capacity (41). In individuals with end-stage CKD, the levels of circulating total and specific miRNAs are generally lower than those in patients with milder forms of renal impairment or normal renal function (42). In our cohort, the observed increase in miR221 expression after renal transplantation was consistent with the findings of other studies. The Increase in miR221 levels was the only variable associated with improved LV diastolic function. These findings suggest that miR-221 is a potential therapeutic target for CKD-related cardiac fibrosis. Additionally, it may be useful as a biomarker in patients with heart failure and preserved LVEF, in which diastolic dysfunction is one of the main pathophysiological features.

Previous studies have demonstrated a relationship between FGF23 levels and hypertension (43). In this population, we observed that patients with higher pre-KT FGF23 levels were more likely to have post-transplant hypertension. However, the linking mechanisms have not been elucidated. In patients with CKD undergoing hemodialysis, FGF23 promotes peripheral vascular and aortic calcification, in addition to carotid atherosclerosis. Calcification mainly affects the medial layer, which reduces vascular elasticity (44). These vascular phenomena are closely related to hypertension and may contribute to post-KT hypertension.

Finally, it was evident that LA reverse remodeling was of lower magnitude than that observed in the LV. Additionally, patients with persistent LA dilation had worse NYHA scores. One of the main determinants of LA volume is LV diastolic function; however, LV diastolic function was similar between different degrees of LA dilatation. This finding suggests that LA abnormalities are responsible for a lower functional status. The LA plays a crucial hemodynamic role by coupling continuous blood flow from the pulmonary bed to the LV, which has pulsatile dynamics (45). Manifestations associated with LA dysfunction range from mild exercise intolerance to pulmonary hypertension, pulmonary edema, and right-sided heart failure, as observed in stiff LA syndrome (46). The minor reversibility of LA abnormalities after KT may suggest a non-return point for the structural damage induced by CRS.

## 5 Limitations

Although diabetes is a common etiology of CKD, in our media a small number of patients with diabetic nephropathy underwent KT. Consequently, to reduce the population heterogeneity, we excluded patients with diabetes. Therefore, these results apply only to individuals without diabetes.

## 6 Conclusions

This study demonstrated that in young patients without diabetes mellitus or other comorbidities, cardiovascular abnormalities due to CKD were almost completely reversed after kidney transplantation. The role played by biomarkers such as FGF23 and PIP helps us to understand the complex pathophysiology of the cardiorenal axis in more detail. The advent of new molecules, such as miR-221, provides essential information from a pathophysiological point of view, in addition to its potential as a biomarker for cardiorenal syndrome and other cardiovascular or renal diseases. The anti-fibrotic effects of miR-221 and its potential therapeutic role in LV diastolic dysfunction warrant further investigation.

## Data Availability

The raw data supporting the conclusions of this article will be made available by the authors, without undue reservation.
